# Magnesium and Vascular Calcification in Chronic Kidney Disease: Current Insights

**DOI:** 10.3390/ijms25021155

**Published:** 2024-01-18

**Authors:** Shari J. Zaslow, Gustavo H. Oliveira-Paula, Wei Chen

**Affiliations:** 1 Department of Medicine, Nephrology Division, Albert Einstein College of Medicine, Bronx, NY 10461, USA; 2 The Robert Larner, M.D. College of Medicine, University of Vermont, Burlington, VT 05405, USA; 3 Department of Molecular Pharmacology, Albert Einstein College of Medicine, Bronx, NY 10461, USA; 4 Wilf Family Cardiovascular Research Institute, Albert Einstein College of Medicine, Bronx, NY 10461, USA; 5 Department of Developmental and Molecular Biology, Albert Einstein College of Medicine, Bronx, NY 10461, USA

**Keywords:** chronic kidney disease, dialysis, magnesium, magnesium supplementation, cardiovascular disease, vascular calcification, calciprotein particles, vascular smooth muscle cell, osteogenic differentiation, hydroxyapatite, mineralization

## Abstract

Magnesium (Mg) plays crucial roles in multiple essential biological processes. As the kidneys are the primary organ responsible for maintaining the blood concentration of Mg, people with chronic kidney disease (CKD) may develop disturbances in Mg. While both hyper- and hypomagnesemia may lead to adverse effects, the consequences associated with hypomagnesemia are often more severe and lasting. Importantly, observational studies have shown that CKD patients with hypomagnesemia have greater vascular calcification. Vascular calcification is accelerated and contributes to a high mortality rate in the CKD population. Both in vitro and animal studies have demonstrated that Mg protects against vascular calcification via several potential mechanisms, such as inhibiting the formation of both hydroxyapatite and pathogenic calciprotein particles as well as limiting osteogenic differentiation, a process in which vascular smooth muscle cells in the media layer of the arteries transform into bone-like cells. These preclinical findings have led to several important clinical trials that have investigated the effects of Mg supplementation on vascular calcification in people with CKD. Interestingly, two major clinical studies produced contradictory findings, resulting in a state of equipoise. This narrative review provides an overview of our current knowledge in the renal handling of Mg in health and CKD and the underlying mechanisms by which Mg may protect against vascular calcification. Lastly, we evaluate the strength of evidence from clinical studies on the efficacy of Mg supplementation and discuss future research directions.

## 1. Introduction

Magnesium (Mg) was reportedly first isolated by Sir Humphry Davy in the early 19th century from the Roman medicinal powder known as “magnesia alba”, which is a byproduct in the production of saltpeter [1,2,3,4]. Throughout the decades, investigations by innumerable researchers have yielded knowledge about the crucial role of this mineral in life and disease. Today, we know that Mg is the second-most-abundant intracellular cation in the human body. Mg is essential for a multitude of biological functions such as coagulation, muscle contraction, and cardiac electrical activity. On the molecular level, Mg is a cofactor in enzymatic reactions and stabilizes the negative charges on DNA. Mg is also necessary for the production and stabilization of ATP. Thus, any downstream reaction that requires ATP can also be thought of as requiring Mg. It is estimated that over 300 enzymatic reactions require Mg [5,6,7]. Piovesan et al. found that out of the entire number of proteins translated in humans, 3751 have potential Mg binding sites [8,9].

In being crucial for so many steps of basic life sustainment, it can easily be seen how perturbations in the level of Mg can lead to health consequences. One of the many disease processes in which Mg is implicated is chronic kidney disease (CKD). CKD has a global prevalence of 9.1%, equating to 700 million cases [10]. In the United States alone, it is estimated that 37 million people are living with CKD, costing Medicare $87.2 billion annually [11]. The leading cause of death in CKD patients is cardiovascular disease. In 2021, approximately 40–45% of deaths among people with end-stage kidney disease were attributed to sudden cardiac death [12]. Vascular calcification is a significant risk factor for cardiovascular morbidity and mortality in this population [13,14,15]. While vascular calcification is not unique to people with CKD, people with CKD have accelerated disease progression and a larger disease burden than the general population [16,17].

In this narrative review, we discuss literature pertaining to the role of Mg in the pathogenesis of vascular calcification in CKD. Specifically, we highlight the current understanding on the renal handling of Mg in health and CKD, potential mechanisms regarding the role of Mg in the pathogenesis of vascular calcification, and whether Mg supplementation is effective for attenuating vascular calcification.

## 2. Mg Homeostasis

### 2.1. Magnesium Homeostasis in Health

It is estimated that the human body contains 24–28 g or 0.4 g/kg of Mg. Mg is almost entirely stored intracellularly in bone and soft tissue. Only 1% of total Mg resides in the extracellular compartment of the blood, 20–30% of which is bound to albumin or other molecules such as phosphate, citrate, and bicarbonate [5,7,18,19,20]. The normal serum Mg level is about 1.8 to 2.2 mg/dL, and this reference range may vary slightly from one lab to another [21]. While Mg is a ubiquitous element that is easily found in most foods, it is most abundant in dark leafy green vegetables, legumes, and grains [22,23]. 

Once consumed, Mg is absorbed through the intestinal brush border via passive paracellular diffusion through tight junctions and actively via non-selective divalent cation transporters with alpha kinase activity—TRPM 6 and 7 (transient receptor potential melastatin-like types 6 and 7). TRPM6 and 7, also known as channel kinase 2 (Chak 2), exhibit serine/threonine kinase activity at their C-terminuses [24,25,26,27]. While TRPM7 is found in all body tissues, TRPM6 is mainly expressed in the intestines and kidneys [28,29,30]. Intracellular Mg is thought to be transported into the blood by CNNM4 (Cyclin and CBS domain Divalent Metal Cation Transport Mediator 4), a basolateral Mg/sodium antiporter found in the intestinal epithelia [31,32].

**Figure 1:**
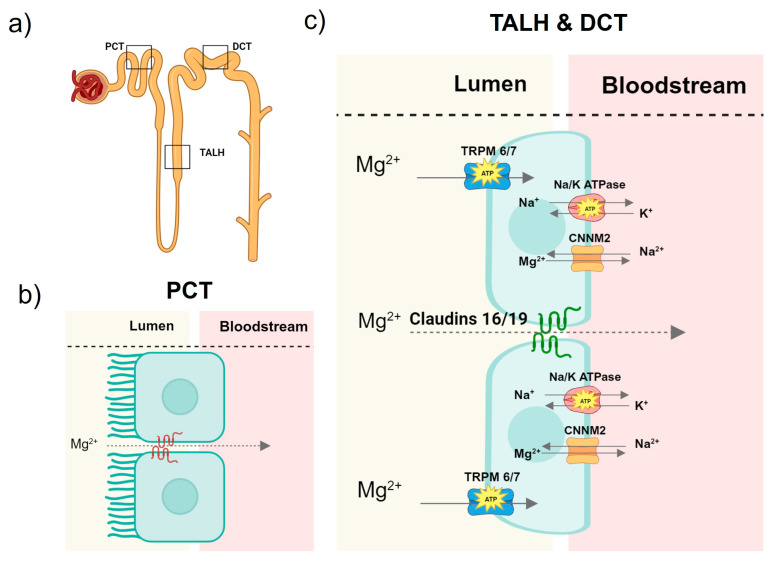
Magnesium reabsorption in the nephron. (**a**) Outline of the key sections of the nephron involved in Mg reabsorption. (**b**) At most one-third of free Mg is reabsorbed paracellularly in the proximal convoluted tubule (PCT). The specific proteins involved in this paracellular process are not yet known. (**c**) The majority of filterable Mg is actively reabsorbed in the thick ascending loop of Henle (TALH) via a divalent cation channel transient receptor potential melastatin 6/7 (TRPM6/7) and paracellularly with claudins 16/19. Once inside the cell, Mg is then transported into the bloodstream via a Mg/sodium antiporter, Cyclin, and the CBS domain Divalent Metal Cation Transport Mediator 2 (CNNM2) by utilizing the electrochemical gradient of sodium generated by the sodium/potassium ATPase. Mg transport in the distal convoluted tubule (DCT) is thought to be the same as in the TALH.

The kidneys are crucial for maintaining a normal blood Mg concentration. At most, one-third of filterable magnesium is reabsorbed in the proximal convoluted tubule composed of cuboidal cells with a brush border [33] (Figure 1). In the proximal tubule, Mg is reabsorbed via a paracellular pathway, though the specific proteins and mechanisms at play are yet to be elucidated [24,33,34,35]. 

In the thick ascending loop of Henle lined with squamous cells [33], approximately 60% of filterable Mg is reabsorbed. Here, Mg, along with other cations, is driven through tight junctions of the tubular epithelium in a paracellular pathway that is mediated by concentration gradients and claudins. Claudins are a family of integral membrane proteins [24,33,35,36]. The expression of claudins in the tight junctions facilitates the transport of cations. Homozygous mutations of claudins 16 and 19 cause familial hypomagnesemia with hypercalciuria and nephrocalcinosis [37]. Additionally, TRPM6/7 actively transports Mg into the luminal cells. The movement of Mg transcellularly is made possible by a Mg/sodium antiporter, CNNM2, which moves Mg from the intracellular space to the bloodstream. The function of this antiporter is made possible by the sodium/potassium ATPase that reduces the intracellular sodium concentration, creating an electrochemical gradient, which then allows sodium to move back into the cell in exchange for Mg [24,36,38]. Finally, the remaining Mg is reabsorbed across the apical membrane in the distal convoluted tubule using the same mechanisms as those in the thick ascending loop of Henle [34].

Unlike calcium, there is no known hormone that is directly responsible for Mg homeostasis. Mg homeostasis is largely maintained by influx via the bowel and reabsorption by the kidneys, balanced with efflux via utilization in biological processes and incorporation into the bone and soft tissue [35,39]. A few studies have identified stimuli associated with changes in TRPM 6/7 expression. TRPM7 was shown to be downregulated by high concentrations of intracellular and extracellular Mg as well as Mg-ATP [25,40]. This finding is supported by a clinical observation, in which higher Mg consumption was associated with lower intestinal Mg absorption [41]. In vitro studies have also identified compounds that influence the expression of TRPM6 in kidney cells including immunosuppressant drugs (e.g., cyclosporine, sirolimus, tacrolimus), 17-β estradiol [41,42,43,44,45,46], iloperidone, and ifenprodil [47].

### 2.2. Mg Disturbances in CKD

In CKD, normal Mg balance is disrupted, resulting in either hyper- or hypomagnesemia. As kidney function declines, less Mg in the blood is filtered through the glomeruli to be excreted, resulting in hypermagnesemia. The prevalence of hypermagnesemia increases as kidney function declines: 2.3% in patients with CKD stages 1–3 and 14.4% in stages 4 and 5 [48]. The treatment of hyperphosphatemia also contributes to hypermagnesemia. For example, sevelamer, a non-absorbed oral phosphate binder, increases the blood concentration of Mg by increasing fatty acid delivery to the intestines and by sequestering bile salts [49].

Patients with CKD may also develop hypomagnesemia due to tubular dysfunction with impaired renal Mg reabsorption and side effects from medications. In a Japanese cohort (n = 5126) [48], the prevalence of hypomagnesemia was estimated to be 15% across all stages of CKD, independent of diuretic use and the variation in albumin levels. In the same study, 114 patients with a median estimated glomerular filtration rate (eGFR) of ~35 mL/min/1.73 m^2^ were randomized to either Mg oxide treatment (starting at 330 mg/day and titrated to achieve a serum Mg level of between 2.5 and 3 mg/dL) or used as controls. In this subgroup, a higher level of proteinuria was associated with greater urinary Mg excretion at baseline, and this relationship was mediated by urinary tubular markers. Compared to controls, Mg oxide significantly increased serum Mg levels after one year, but only among participants with a urinary protein-to-creatinine ratio of less than 0.3 g/g, while there was no change in serum Mg in those with greater levels of proteinuria. These findings suggest that hypomagnesemia is a consequence of tubular injury.

In addition to tubular dysfunction, there are many factors that predispose CKD patients to hypomagnesemia. These factors include the use of proton pump inhibitors, diuretics, and potassium binders and a low dialysate Mg concentration. The use of proton pump inhibitors among CKD patients is prevalent. In a French cohort (n = 3023), it was estimated that 32% of people with CKD stages 2–5 were on a proton pump inhibitor [50]. Proton pump inhibitors may lower the Mg level in the blood by increasing the pH in the intestinal lumen. The change in pH can then decrease Mg absorption by altering the expression of paracellular claudins and the transcellular transporter TRPM 6/7 [51]. A meta-analysis of observational studies showed that the use of proton pump inhibitors was associated with ~1.7 times higher odds of having hypomagnesemia [52].

Other common medicinal culprits of hypomagnesemia are diuretics and potassium binders. By blocking the sodium/potassium/chloride co-transporter, loop diuretics reduce the substrate required for the sodium/potassium ATPase and subsequently remove the electrochemical gradient for the sodium/Mg antiporter, thus inhibiting the transcellular reabsorption of Mg (Figure 1) [53,54]. Thiazide diuretics may downregulate TRPM6, thus preventing renal magnesium reabsorption [55]. Patients with advanced or end-stage kidney disease may develop life threatening hyperkalemia. To prevent this, oral potassium binders, such as patiromer, are used. Patiromer is a crosslinked, non-absorbed polymer that uses calcium as a counter ion for the exchange of potassium in the colon [56]. Patiromer can also bind to Mg in the colon to decrease its absorption [57]. In a clinical trial, the most common adverse effect attributable to patiromer was hypomagnesemia, which was experienced by 7.2% of the study participants [58].

In patients with end-stage kidney disease, renal replacement therapy becomes necessary. Two types of renal replacement therapy are hemodialysis and peritoneal dialysis to remove waste and fluid. In hemodialysis, blood is filtered through a dialyzer against dialysate in a countercurrent fashion. In peritoneal dialysis, dialysate is infused into the abdominal cavity, while waste and fluid are removed from the blood into the dialysate using the abdominal peritoneum as a filter. In patients with end-stage kidney disease, the serum Mg level is largely determined by Mg intake and the amount of Mg removed by dialysis. In the early days of dialysis, hypermagnesemia was a concern, because it was thought to suppress parathyroid hormone and contribute to adynamic bone disease [59]. Over the past decades, the commonly used concentration of dialysate Mg decreased from approximately 1.5 mEq/L in the 1970s to 1.0 mEq/L [60], leading to increased removal of total body Mg and an increased prevalence of hypomagnesemia in dialysis patients. 

Symptoms of hypermagnesemia range from nausea and dizziness to flaccid paralysis and cardiac arrest [61], whereas symptoms of hypomagnesemia may include arrythmia, an altered mental status, and tremors [62]. While both hyper- and hypomagnesemia can be life threatening, hypomagnesemia may have more consequences over time. In the following sections, we detail the molecular mechanisms of vascular calcification in CKD and the role of Mg in vascular calcification. 

## 3. Vascular Calcification in CKD

Vascular calcification is the pathological deposition of calcium and phosphate in the walls of blood vessels. It is accelerated in patients with CKD, and its prevalence increases as kidney function declines [63]. In a cohort of 367 patients in the United States with end-stage kidney disease who were newly started on hemodialysis, 50–60% had calcification in the coronary arteries or thoracic aorta [64]. Patients with CKD may develop vascular calcification in both the intimal and medial layers of vessel walls. Calcification in the intimal layer, or intimal calcification, is associated with atherosclerosis and narrowing of the blood vessel lumen and contributes to ischemic heart disease [65]. Calcification in the media layer, or medial calcification, is characterized by diffuse depositions of calcium and phosphate and is associated with arterial stiffness, systolic hypertension, and left ventricular hypertrophy [66,67]. Both intimal and medial calcification contribute to the high mortality rate in people with CKD.

The pathogenesis of vascular calcification is cell-mediated. In the arteries, endothelial cells constitute the inner cellular lining, while most cells in the medial layer are vascular smooth muscle cells (VSMCs). VSMCs normally have a contractile phenotype, but under pro-calcifying conditions, they can transform into osteoblast-like cells in a process called osteogenic differentiation. The osteogenic differentiation of VSMCs is characterized by decreased expression of smooth-muscle-cell-specific contractile proteins, increased expression and activity of the major osteogenic transcription factors such as runt-related transcription factor-2 (*Runx-2*), and increased expression of bone markers such as alkaline phosphatase [68,69,70,71,72]. Alkaline phosphatase catalyzes the degradation of inorganic pyrophosphate—a major inhibitor of calcium phosphate nucleation [73]. The osteoblast-like cells then produce a collagen matrix and form calcium- and phosphorus-rich matrix vesicles that are capable of initiating mineralization in the vessel walls [74,75]. Although VSMCs are the major cell type involved in vascular calcification, endothelial cells in the arterial intima can regulate the osteogenic differentiation of VSMCs by releasing factors such as nitric oxide and endothelial microvesicles [76,77,78,79,80].

Patients with CKD often have concomitant diabetes mellitus, hyperlipidemia, and disordered metabolisms of calcium and phosphate [81]. All of these may contribute to vascular calcification. The biological effects of hyperglycemia and hyperlipidemia on vascular calcification are likely mediated via inflammation involving cytokines and oxidative stress [82,83]. Hyperglycemia leads to the accumulation of advanced glycation end products that upregulate transcription factor nuclear factor-κB (NF-κB) and its target genes [84] and promote the post-translational modification of proteins via *O*-linked b-N-acetylglucosaminylation to modulate osteochondrogenesis [85,86]. Calcium and phosphate are integral components of hydroxyapatite (i.e., calcium phosphate crystals). Mediated by sodium–phosphate transporters, a high phosphate diet upregulates *Runx-2* and induces vascular calcification in mice with 5/6 nephrectomy-induced CKD [87]. Elevated calcium enhances the activity of these sodium–phosphate transporters and blocks L-type calcium channels, which inhibit mineralization in VSMCs [88,89].

**Figure 2:**
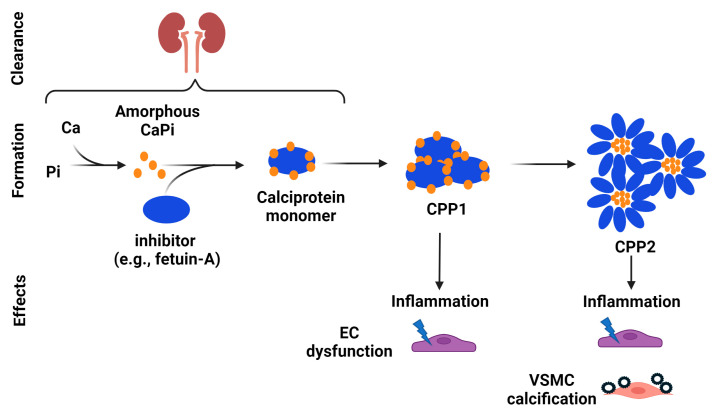
The potential role of calciprotein particles in vascular calcification. Calciprotein particles (CPPs) are nanoparticles composed of calcium phosphate crystals and calcification inhibitors, such as fetuin-A and albumin. There are three types of CPPs—calciprotein monomers, primary CPPs (CPP1), and secondary CPPs (CPP2). Calciprotein monomers are cleared in the kidneys. With decreased renal clearance of calcium and phosphate in CKD, calciprotein monomer production increases. Together with decreased renal excretion of calciprotein monomers, excess calciprotein monomers coalesce to form CPP1. CPP1 then undergoes Ostwald ripening and transition into crystalline CPP2. At high levels, CPP1 and CPP2 contribute to the pathogenesis of vascular calcification by inducing vascular inflammation via the NF-κB axis and inflammasome activation and decreasing endothelial cell (EC) function. The exposure of vascular smooth muscle cells (VSMCs) to CPP2 leads to pronounced and concentration-dependent calcification.

The effects of calcium and phosphate on vascular calcification may also be mediated via calciprotein particles (CPPs). CPPs are nanoparticles composed of calcium phosphate crystals and calcification inhibitors such as fetuin-A and albumin (Figure 2) [90]. There are three types of CPPs–calciprotein monomers, primary CPPs (CPP1), and secondary CPPs (CPP2). Calciprotein monomers are the predominant circulating form and are a physiological transporter of excess calcium and phosphate [91,92,93]. Calciprotein monomers are cleared in the kidneys [93,94]. With decreased renal clearance of calcium and phosphate in CKD, calciprotein monomer production increases. Together with decreased renal excretion of calciprotein monomers, excess calciprotein monomers coalesce to form CPP1. CPP1 then undergoes Ostwald ripening and transition into crystalline CPP2 [95,96]. CPPs mediate the transport and clearance of calcium and phosphate in the blood and are normally a potent mechanism to prevent soft tissue calcification; however, at high levels, CPP1 and CPP2 contribute to the pathogenesis of vascular calcification by inducing vascular inflammation via the NF-κB axis and inflammasome activation and decreasing endothelial cell function [97,98,99,100,101,102,103]. The exposure of VSMCs to CPP2 leads to pronounced and concentration-dependent calcification, and the calcification is ameliorated by the suppression of tumor necrosis factor-α [100].

Despite the high prevalence and detrimental effects of vascular calcification in CKD patients, there is no established treatment for vascular calcification. Given the effects of phosphate on vascular calcification in animal studies [87], current therapeutic strategies focus on lowering serum phosphate [68,69,87]. However, clinical trials have failed to show that lowering serum phosphate is sufficient to treat or slow vascular calcification [104,105,106,107]. Thus, there is an urgent need to identify new therapeutic targets and strategies, and studying Mg and vascular calcification holds the potential to shift the current therapeutic paradigm.

## 4. Epidemiology for Mg and Vascular Calcification in CKD

In a large cohort of patients on hemodialysis (n = 142,555), there were J-shaped relationships of serum Mg with both cardiovascular and non-cardiovascular mortality, with a serum Mg level of around 2.8 mg/dL being associated with the lowest mortality rate [108]. The association was most striking for hypomagnesemia and cardiovascular mortality. Compared to those with serum Mg concentrations of between 2.8 and <3.1 mg/dL, patients on hemodialysis with a serum Mg concentration of <2.3 mg/dL had 1.8 times higher odds of cardiovascular mortality. This finding was further supported by a recent meta-analysis [109]. Hypomagnesemia is certainly a risk factor for cardiac arrythmia, but this relationship could be partly explained by the relationship between Mg and vascular calcification.

Generally, hypomagnesemia is associated with greater vascular calcification [64,110,111]. In a cross-sectional study of 80 patients on peritoneal dialysis in Canada, hypomagnesemia was associated with greater abdominal aortic calcification, independently of age, serum phosphate, parathyroid hormone, cholesterol, smoking history, and diabetes [110]. In the aforementioned cohort of 367 patients from the United States who had been on hemodialysis for less than 6 months [64], hypomagnesemia was associated with calcification in the coronary arteries and thoracic aorta in those without diabetes, independently of demographics, smoking history, and levels of serum calcium, phosphate, parathyroid hormone, and fetuin-A. Interestingly, the opposite relationship was found among diabetic patients, in which hypermagnesemia was associated with aortic calcification [64]. These findings suggest that the protective effect of Mg on calcification may be independent of the metabolic abnormalities associated with diabetes, and the factors inducing vascular calcification in diabetes may mask any effects of Mg associated with the prevention of calcification.

## 5. Mg and Vascular Calcification: In Vitro and Animal Studies

**Figure 3:**
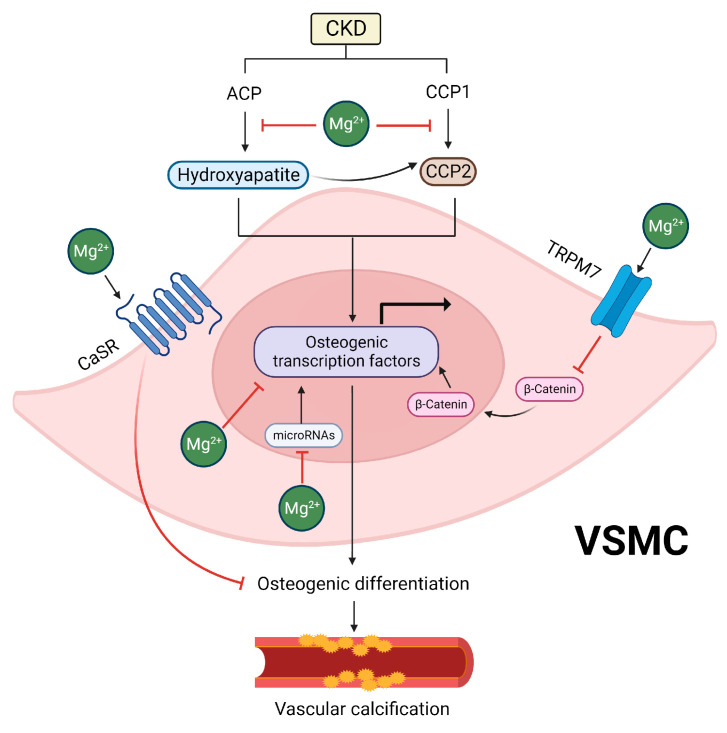
Overview of the potential mechanisms underlying the inhibitory effects of magnesium on vascular calcification. In chronic kidney disease (CKD), imbalances in calcification inhibitors and inducers promote the formation of amorphous calcium/phosphate particles (ACPs). These particles nucleate and mature into hydroxyapatite crystals, which are crucial for the osteogenic differentiation of vascular smooth muscle cells (VSMC). Mg demonstrates inhibitory effects on hydroxyapatite formation, and studies suggest it also hinders vascular calcification by influencing the maturation of calciprotein particles 2 (CPP2), which also induces the transformation of VSMCs into an osteogenic phenotype, ultimately contributing to vascular calcification. In addition, since CPP2 consists of a crystalline hydroxyapatite core, it is possible that the prevention of hydroxyapatite crystal formation by Mg might also contribute, at least in part, to its effects on CPP2-mediated vascular calcification. The anti-calcifying effects of Mg also involve decreased expression of osteogenic transcription factors and the prevention of the loss of calcification inhibitors. Mechanistically, the divalent cation channel transient receptor potential melastatin 7 (TRPM7), a major Mg channel in VSMCs, plays an important role in the development of calcification. Mg uptake by TRPM7 inhibits the Wnt/β-catenin signaling pathway, which is known to regulate genes promoting VSMC osteogenic differentiation. Additionally, Mg prevents high phosphate-induced VSMC osteogenic differentiation by activating the calcium-sensing receptor (CaSR), which acts as a calcimimetic to reduce vascular calcification. MicroRNA modulation is another potential mechanism, with Mg counteracting the disrupted expression of specific microRNAs (i.e., 30b, 133a, and 143) promoted by high phosphate levels, contributing to the inhibition of vascular calcification.

The observations in epidemiological studies are generally in line with the protective effects of Mg on vascular calcification reported in cellular and animal models [112,113,114,115,116,117,118,119]. When added to culture media enriched with phosphate and calcium, Mg attenuated calcification in vitro in human, bovine, and rat VSMCs [117,118,119]. In addition, Mg showed protective effects on calcification in rat aortic rings exposed to high phosphate levels and in animal models of CKD, such as adenine-induced CKD rats and partially nephrectomized rats [112,113,114,115,116]. In the section below, we discuss the protective effects of Mg against vascular calcification via its direct effects on hydroxyapatite and CPP formation and the osteogenic differentiation of VSMCs, as well as its indirect effects (Figure 3). We also summarize the evidence from animal studies.

### 5.1. Inhibitory Effects of Mg on Hydroxyapatite and CPP Formation

The inhibitory effects of Mg on vascular calcification may stem from its impacts on hydroxyapatite and CPPs, which are both major drivers of vascular calcification [100,101]. In CKD, decreased levels of inhibitors and simultaneous increases in the levels of inducers of calcification (e.g., hyperphosphatemia, hypercalcemia, and oxidative stress) promote the formation of amorphous calcium/phosphate particles [120]. The initiation of vascular calcification occurs through the nucleation and maturation of amorphous calcium/phosphate particles into hydroxyapatite crystals, which has been shown to be essential for VSMC osteogenic differentiation [121]. Interestingly, Mg seems to exert inhibitory effects on hydroxyapatite formation through different mechanisms [122,123,124,125]. One of the proposed mechanisms relies on the fact that Mg can replace calcium in hydroxyapatite formation, promoting the generation of Mg-containing whitlockite [123,124,125]. Notably, whitlockite is occasionally detected in the calcified arteries of patients with CKD and uremic rats, and it has been identified as less pathogenic when compared to hydroxyapatite [126,127,128,129]. A different mechanism was proposed by Braake et al., who found that Mg supplementation limits VSMC calcification by preventing hydroxyapatite crystal formation in the extracellular space via the inhibition of crystal nucleation [122]. This effect was found to be independent of TRPM7, a major Mg channel in VSMCs. While the authors did not evaluate the exact mechanisms by which Mg inhibits crystal nucleation, which prevents hydroxyapatite formation, this effect may be related to the fact that Mg limits the hydrolysis of extracellular ATP, which is necessary for hydroxyapatite nucleation [130].

In addition to preventing hydroxyapatite formation, studies suggest that Mg inhibits vascular calcification by affecting CPP2 maturation. Notably, Mg does not seem to hinder the initial formation of CPP1 from supersaturated concentrations of calcium and phosphate [102]. However, it prevents their maturation into crystalline CPP2 [102,131], which has a lower solubility and a tendency to crystallize (Figure 2) [132]. Once CPP2 has fully maturated, Mg seems to be ineffective for preventing CPP2-induced VSMC mineralization and the expression of osteogenic proteins [102]. Given that CPP2 maturation may occur before the trans-differentiation of VSMCs into an osteoblast-like phenotype, the prevention of CPP2 maturation by Mg would result in the decreased expression of osteogenic genes, preserving VSMCs in their contractile phenotype [123]. While the exact mechanisms by which Mg prevents CPP2 maturation remain to be determined, it is possible that the prevention of hydroxyapatite crystal formation, as discussed above, might contribute, at least in part, to these effects. This is because CPP2 consists of a crystalline hydroxyapatite core [133], and therefore, the inhibition of hydroxyapatite formation by Mg might consequently impair CPP2 formation, ultimately reducing vascular calcification.

### 5.2. Inhibitory Effects of Mg on the Osteogenic Differentiation of VSMCs

Apart from its impacts on hydroxyapatite and CPP formation, studies have shown that Mg also affects vascular calcification at the intracellular level [121,134]. As previously discussed, the transformation of VSMCs into an osteogenic phenotype is recognized as a significant contributor to the development and advancement of vascular calcification [135]. Mg may inhibit vascular calcification by modulating the pathways related to VSMC osteogenic differentiation [121,134]. Indeed, the anti-calcifying effects of Mg are associated with decreased expression of osteogenic transcription factors, such as bone morphogenetic protein-2 (BMP-2), *Runx-2*, and *Msh homeobox 2*, and they prevent the loss of calcification inhibitors such as matrix Gla protein (MGP), osteopontin, and BMP-7 [121]. Additionally, Mg supplementation counteracts the expression of genes associated with matrix mineralization, such as osteocalcin and alkaline phosphatase [134].

Different mechanisms have been proposed to elucidate how Mg directly prevents VSMC osteogenic differentiation [118,136,137,138,139]. One potential mechanism involves TRPM7, the major Mg channel in VSMCs [140]. Interestingly, treatment with 2-aminoethoxydiphenyl borate, a non-selective TRPM7 blocker, counteracted the protective effects of Mg supplementation in rat VSMCs exposed to high phosphate levels [138]. In this study, Mg decreased the expression of the pro-osteogenic protein osteocalcin and increased the expression of the anti-calcification protein MGP in rat VSMCs exposed to high phosphate levels, and these effects were counteracted by TRPM7 inhibition. In addition, this study evaluated VSMCs and aortic sections harvested from mice with high or low intracellular Mg levels that were exposed to a calcification medium. The authors found that osteocalcin expression was increased in VSMCs exposed to a calcification medium and in vessels from mice with low Mg levels but not from those with high Mg levels. Furthermore, osteopontin was increased in mice with high Mg levels, but not in those with low Mg levels [138]. Accordingly, another study using 2-aminoethoxydiphenyl borate in human VSMCs also found that TRPM7 inhibition prevented the protective effects of Mg on vascular calcification [118].

In line with these findings, the knockdown of TRPM7 with a small interfering RNA prevented the inhibitory effects of Mg on the calcification of human VSMCs [137]. Mechanistically, studies have shown that Mg uptake by TRPM7 in VSMCs inhibits the Wnt/β-catenin signaling pathway, which is known to modulate the transcription of genes that promote VSMC osteogenic differentiation and vascular calcification [137]. Indeed, Montes de Oca et al., observed upregulation of the Wnt/β-catenin pathway in VSMCs exposed to high phosphate, as evidenced by the translocation of β-catenin into the nucleus, increased expression of the frizzled-3 gene, and the downregulation of the Dkk-1 gene—a specific antagonist of the Wnt/β-catenin signaling pathway [137]. Conversely, Mg supplementation hindered the activation of the Wnt/β-catenin signaling pathway induced by a high phosphate concentration in VSMCs. Additionally, the silencing of TRPM7 using siRNA led to the activation of the Wnt/β-catenin signaling pathway in those cells [137]. Taken together, these findings suggest that the protective effects of Mg on vascular calcification are, at least in part, mediated by TRPM-7-dependent inhibition of the Wnt/β-catenin signaling pathway, ultimately preventing VSMC osteogenic differentiation.

In addition to TRPM7, studies have shown that Mg prevents high-phosphate-induced VSMC osteogenic differentiation and calcification by activating the calcium-sensing receptor (CaSR) [136]. CaSR is a G-protein-coupled receptor that controls the ionic calcium influx in VSMCs, and a reduction in its expression in the vasculature has been associated with vascular calcification [141]. It is proposed that Mg could work as calcimimetic/gatekeeper that activates CaSR and prevents the influx of ionic calcium [142]. Consistent with this idea, Alesutan et al., found that the inhibitory effects of Mg on VSMC osteogenic differentiation and calcification were counteracted by the CaSR antagonist NPS-2143 or by silencing of the *CaSR* gene [136]. Using human VSMCs, the authors observed that Mg promoted the upregulation of *CaSR* mRNA expression in a dose-dependent manner. Moreover, the inhibitory impact of Mg on high-phosphate-induced calcium deposition and the mRNA expression of osteogenic markers was mimicked by the CaSR agonist gadolinium chloride, while additional treatment with the CaSR antagonist NPS-2143 or silencing of the *CaSR* gene in VSMCs reversed these effects. Mg also mitigated the osteogenic transformation of VSMCs induced by hydroxyapatite particles. In mice, high-dose cholecalciferol treatment promoted vascular calcification and elevated aortic mRNA expression of osteogenic markers (*Msx2*, *Cbfa1*, *Alpl*), collagen type I (*Col1a1*), collagen type III (*Col3a1*), and fibronectin (*Fbn*). These effects were alleviated by treatment with Mg. Furthermore, the increased aortic *CaSR* mRNA expression observed in cholecalciferol-treated mice was further enhanced by Mg [136]. Altogether, these findings suggest that Mg supplementation may inhibit vascular calcification, at least in part, by activating CaSR.

Modulation of the expression of specific microRNAs (miR) is another potential mechanism by which Mg inhibits vascular calcification [139]. In this respect, Louvet et al. found that the beneficial impact of Mg on high-phosphate-induced vascular calcification in VSMCs is associated with the re-established expression of miR-30b, miR-133a, and miR-143, which were found to be downregulated by high phosphate levels [139]. Consistent with these observations, the authors also found that a high phosphate concentration disrupted the expression of osteogenesis markers related to these miRs, such as Smad1 and Osterix, whereas Mg reversed this effect.

### 5.3. Indirect Effects of Mg Supplementation

Dietary Mg may have indirect protective effects on vascular calcification via the binding of Mg to phosphate in the intestinal lumen, thus decreasing phosphate uptake through the sodium phosphate cotransporter IIb in enterocytes [121]. Mg may also indirectly affect vascular calcification by improving endothelial function. Indeed, studies have shown that Mg increases nitric oxide levels and prevents NF-κB activation and cytokine and chemokine production in endothelial cells in vitro [143,144,145]. As discussed above, Mg also exerts inhibitory effects on CPP [102], which is known to promote endothelial dysfunction and disrupt the formation and metabolism of endothelial-cell-derived factors [98,99,103]. Given that endothelial-cell-derived factors can affect vascular calcification and the osteogenic differentiation of VSMCs [76,78,146], it is reasonable to speculate that the improvement of endothelial function by Mg might have beneficial implications for vascular calcification.

### 5.4. Effects of Mg on Vascular Calcification In Vivo

The effects of Mg on vascular calcification shown by in vitro studies seem to also be relevant in vivo [112,113,114,115,147]. A high-Mg diet (0.2% Mg) limited calcitriol-induced vascular calcification in the aortas, iliac arteries, and carotid arteries of Sprague–Dawley rats with adenine-induced CKD [115]. In line with the mechanisms discussed above, these findings were associated with the recovery of the vascular TRPM7 expression levels by Mg, which had been reduced by calcitriol [115]. In another study, Diaz-Tocados et al., found that dietary Mg supplementation (0.3–1.1% Mg) improved renal function, attenuated vascular calcification and the expression of osteogenic markers, and reduced the mortality rate of 5/6 nephrectomized rats [114]. Interestingly, the authors observed that Mg administered intraperitoneally (30 mg/kg) also decreased vascular calcification, suggesting that the effects of Mg on vascular calcification are not limited to its actions as an intestinal phosphate binder. Consistent with these results, other studies also observed protective effects of Mg against vascular calcification in adenine-induced CKD rats (diet containing Mg citrate 750 mg/kg) and in partially nephrectomized rats (Mg enriched 0.48% *w*/*w* diet) [112,113].

Mg also limited vascular calcification induced by the inactivation of the *Klotho* gene, a key regulator of the phosphate and calcium balance. *Klotho* knockout mice develop hyperphosphatemia with subsequent severe vascular calcification, and therefore, they have been used as a model of this disorder [148]. Interestingly, Mg supplementation (Mg enriched 0.48% *w*/*w* diet) prevented vascular calcification and the aortic expression of *Runx2* and MGP in *Klotho* knockout mice [147]. These results were associated with reductions in the inflammatory and extracellular matrix remodeling pathways in the aortas of *Klotho*-deficient mice. Altogether, these findings suggest that Mg has a beneficial impact on vascular calcification in vivo, with potential translational applications.

## 6. Mg and Vascular Calcification: Clinical Studies

Following evidence from epidemiological and animal studies, several clinical trials were performed to evaluate the effects of Mg supplementation on vascular calcification in people with CKD. Mg was supplemented either orally in pre-dialysis CKD patients or by increasing the concentration of dialysate Mg for patients with end-stage kidney disease.

There are two major clinical trials that have examined the effects of oral Mg supplementation on the progression of vascular calcification. The first is an open label, randomized, controlled trial conducted in Japan [149]. Patients with CKD stages 3–4 and risk factors for coronary artery calcification (i.e., diabetes mellitus, history of cardiovascular disease, high LDL cholesterol, and smoking) were included. Sixty-three (63) received oral Mg oxide, whereas 60 were assigned to the control group. In the Mg oxide group, patients received 330 mg of Mg oxide (8.3 mmol of elemental Mg) per day, and the dose was adjusted every 1–3 months to achieve serum Mg levels of 2.5–3.0 mg/dL. Controls received “standard therapy for CKD alone”. At baseline, the mean serum Mg level was 2.0 mg/dL in the treatment group and 2.1 mg/dL in controls. After a 2-year follow up period, the serum Mg concentration increased to 2.3 mg/dL within the treatment group, while there was no change in the controls. Coronary artery calcification was quantified by computed tomography. At baseline, the median coronary artery calcification score was 266 in the treatment group and 166 in the controls. An interim analysis demonstrated favorable effects of Mg oxide: in the treatment group, the median increase in the coronary artery calcification score was 11.3%, which was significantly smaller than that of the control group (39.5%). In addition, the proportion of patients with an annualized percentage change in the coronary artery calcification score of 15% was significantly lower for the treatment group compared to the controls. As a result, the study was terminated earlier than planned.

The other study is a randomized, double-blind, placebo-controlled, clinical trial that was conducted in Denmark and Norway [150]. Patients with an eGFR of between 15 and 45 mL/min per 1.73 m^2^ (n = 148) were randomized to receive either oral Mg hydroxide (15 mmol of elemental Mg twice daily) or a matching placebo. At baseline, the mean plasma Mg level was 2.0 mg/dL in both groups, and the median coronary artery calcification scores were 413 and 274 in the Mg and placebo groups, respectively. After a 1 year follow up period, the mean plasma Mg concentration in the treatment group increased to 2.3 mg/dL, which was higher than the placebo group’s concentration of 2.0 mg/dL. Unfortunately, despite the increase in the plasma Mg level, there was no difference in the progression of coronary artery calcification between the treatment and placebo groups. 

It is unclear why the effects of Mg supplementation on coronary artery calcification differ between these two studies. While both studies were well designed and executed, the quality of the second clinical trial was better due to the use of blinding and placebo groups, which minimized the sources of bias. Notably, these two studies varied in terms of their study populations and durations. It is well known that the CKD population in Japan has a better survival rate than that in Western countries. Compared to the rate in Japan, the relative risk of mortality in hemodialysis patients was shown to be 2.8 times higher for Europe and 3.8 times higher for the United States independently of age, gender, race, and comorbid conditions [151]. In addition, in the first clinical trial, the study population was enriched with a risk factor of vascular calcification, with ~75% of participants having the diagnosis of diabetes, while only approximately 40% had diabetes in the second study. The difference in the proportion of participants with diabetes may be important because diabetes is a risk factor for both hypomagnesemia and vascular calcification [152,153]. Lastly, compared to the second study, the first had a greater follow up period, which is important because vascular calcification often takes years to develop.

In patients with end-stage kidney disease who are on dialysis, increasing the dialysate Mg concentration is an important avenue to provide supplementation. We identified two such clinical studies, but both were relatively small in terms of their sample sizes and neither included the measurement of vascular calcification as an outcome. In a pilot study, 25 patients received dialysate with a high Mg concentration (1.5 mEq/L) while 50 received low-dialysate Mg (1.0 mEq/L) [154]. After a 3-year follow up period, patients who received high-dialysate Mg had a 65% reduction in all-cause mortality compared to those who received low-dialysate Mg with a hazard ratio of 0.35 and a wide 95% confidence interval from 0.13 to 0.97. In another study, Bressendorff et al., conducted a single-center, randomized, double-blinded, paralleled group, controlled clinical trial to examine the effects of increasing the dialysate Mg from 1.0 mEq/L to 2.0 mEq/L in 59 patients on hemodialysis [155,156]. After 4 weeks of intervention, the use of high-dialysate Mg increased the serum Mg concentration by 0.9 mg/dL, which is a greater increase than what was achieved in the studies of oral Mg [149,150]. The outcome was serum T_50_, which is the time of transformation from CPP1 to CPP2 ex vivo; [90] T_50_ increased with a high-dialysate Mg concentration, indicating a slower transformation of CPP1 to the more crystalline CPP2.

## 7. Perspectives

The contradictory findings of the two clinical trials on oral Mg supplementation and vascular calcification have resulted in a state of equipoise that underscores the need for further investigation. When designing clinical trials to examine the effects of Mg on vascular calcification, it is worth considering the following four aspects. First, unlike animal models, such as adenine-induced and *klotho*-knocked-out [114,115,147,148], patients with CKD often have multiple concomitant risk factors (e.g., hyperglycemia, hyperlipidemia, and hyperphosphatemia) for vascular calcification. A multi-faceted approach using combination therapy may produce a more desirable outcome than that achieved by targeting one single risk factor, such as Mg.

Second, vascular calcification progresses over time and is often irreversible. In animal studies, Mg was supplemented before the phenotype of vascular calcification developed [114,147]. Mg may be beneficial for preventing vascular calcification, but not for attenuating it once it develops. Thus, future strategies should focus on prevention and on people with early-stage CKD, who have not yet developed vascular calcification or have mild disease.

Third, the current gold standard for the clinical detection and quantification of vascular calcification is computed tomography [157], which only allows for the identification of advanced and macro-calcification, but not micro-calcification. Compared to macro-calcification, micro-calcification (<50 μm) indicates an earlier process involving active inflammation and confers an additional cardiovascular risk [158,159,160,161,162]. ^18^F-sodium fluoride binds to the surface of hydroxyapatite by exchanging hydroxyl ions to form fluoroapatite, and ^18^F-sodium fluoride positron-emission tomography can be combined with computed tomography to detect nascent micro-calcification [162,163]. However, the application of this novel imaging technique has not been widely adapted to clinical trials, particularly those focusing on CKD patients.

Lastly, as Mg may suppress parathyroid hormone and contribute to adynamic bone disease [59], the effects of Mg supplementation on bone health should be evaluated in addition to vascular calcification. In both studies on oral Mg supplementation, there was no change in the parathyroid hormone level, but neither study examined bone health [149,150]. In a study on dialysate Mg [155], the parathyroid hormone level decreased by ~21% after treatment with high-dialysate Mg, reflecting a possible suppression of bone turnover. 

A pragmatic trial is currently underway in Canada to examine the effects of the dialysate Mg concentration on clinical outcomes (clinicaltrials.gov; NCT04079582) [164]. Compared to an explanatory trial, a pragmatic trial evaluates the effectiveness of interventions under real-life conditions, producing results that can be more generalized and readily applied to routine clinical practice. In this study, approximately 25,000 patients have been randomized to a dialysate Mg concentration of either 1.5 mEq/L or ≤1.0 mEq/L for 3–4 years. The primary outcome is a composite of cardiovascular-related hospitalization and all-cause mortality. We anticipate that the findings from this trial will provide significant insights into the optimal and safest dialysate Mg concentration and inform whether adjusting Mg levels in the dialysate will impact the clinical outcomes of CKD patients.

As we await the results of clinical studies, a better understanding of the mechanisms of Mg on vascular calcification will provide valuable insight into therapeutics. For example, it may be important to delineate the mechanism by which Mg inhibits crystal nucleation in the vasculature. Many mechanistic studies of Mg have used an in vitro approach, and whether these signaling pathways remain valid in animal models with a loss or gain of function approach is unknown and warrants investigation. Research on the biological function of CPP in vascular calcification is still in its infancy. Mg may have CPP effects beyond the transformation process from CPP1 to CPP2. Although Mg may not be a panacea for vascular calcification, we are hopeful that research on Mg will contribute to the development of an effective therapeutic approach and ultimately improve the cardiovascular outcomes of people with CKD.

## 8. Conclusions

Mg plays crucial roles in numerous essential biological processes associated with life. Kidneys are the primary organ responsible for maintaining and regulating the concentration of Mg in the blood. In people with CKD, Mg hemostasis is disrupted. Hypomagnesemia is generally associated with greater vascular calcification, which is accelerated and prevalent in this population. Both in vitro and animal studies suggest that Mg is protective against vascular calcification. These findings have led to several clinical trials to investigate the effects of Mg supplementation on vascular calcification in people with CKD. Interestingly, two major clinical studies have produced contradictory findings, creating a state of equipoise that underscores the need for further investigation. In this review, we conducted a comprehensive examination of the findings from both basic science and human studies, incorporating the advances in CPP research, a recently published clinical trial [150], and an ongoing trial on Mg supplementation [164].
